# Multibranch Gold Nanoparticles as Surface-Enhanced Raman Spectroscopy Substrates for Rapid and Sensitive Analysis of Fipronil in Eggs

**DOI:** 10.3390/s19245354

**Published:** 2019-12-05

**Authors:** Haonuan Zhao, Dandan Huang, Shuhua Zhu

**Affiliations:** College of Chemistry and Material Science, Shandong Agricultural University, Tai’an 271000, China; zhaohaonuan@163.com

**Keywords:** surface-enhanced Raman spectroscopy, gold nanoparticles, fipronil, eggs, rapid analysis

## Abstract

A sensitive strategy to rapidly detect fipronil residues in eggs using multibranch gold nanoparticles (AuNPs) as the substrate of surface-enhanced Raman spectroscopy (SERS) was investigated in this study. Under optimized conditions, fipronil molecules preferentially deposited on the multibranch gold nanoparticles with preferential (111) facet-oriented growth due to its low surface energy. This anisotropic growth promoted the increase of SERS “hot spots”, inducing a huge enhancement of Raman signals of the fipronil. An external standard calibration method was employed for quantitative analysis, and the method was validated for linearity, sensitivity, repeatability and recovery. Good linearity were found in the concentration range of 10 ng/L~10 mg/L in fipronil acetone solution (*R^2^* = 0.9916) and 8 × 10^−5^ mg/m^2^ to 0.8 mg/m^2^ on eggshells (*R^2^* = 0.9906), respectively. The recovery rate based on acetone recovered fipronil on eggshells and in egg liquids was 80.13%~87.87%, and 81.34%~88.89%, respectively. The SERS assay was successfully used to monitor fipronil in eggs.

## 1. Introduction

Since the fipronil egg scandal was exposed in August 2017, eggs containing fipronil residue have been found in more than 45 countries. Millions of eggs are recalled from supermarkets, and many poultry farms have suffered economic losses [1]. Except for European countries, Asian areas like South Korea, Taiwan and Hong Kong were also found the fipronil-contaminated eggs. The European Union (EU) reports that the contents of fipronil in eggs and chicken reach up to 0.72 and 0.77 mg/kg, respectively. However, another report has shown that the content of fipronil in Belgian eggs was 1.2 mg/kg in the studied sample [2].

As the first phenylpyrazole insecticide in the world, fipronil (trade name: Regent) is developed by the French company Rhône-Poulenc [3]. Fipronil is toxic to most fish and birds and exerts sublethal effects, including genotoxicity, cytotoxicity and impairing immune function [4]. Also, fipronil and its metabolites can be hardly degraded, and they are all highly toxic to the environment [4]. When exposed to light, fipronil is more sensitive, with a half-life of 770 h at pH 9.0, 114 h at pH 10.0 and 11 h at pH 12.0, but is stable under acid and neutral conditions in water [5]. Fipronil is also highly harmful to crustacean aquatic organisms and bees [6]. Therefore, the Ministry of Agriculture of China issued a public announcement in 2009 to stop the production and sale of fipronil pesticides [7]. A new national standard using liquid chromatography-mass spectrometry (LC-MS) on the determination of fipronil in eggs was announced on 21 June 2018, and took effect after 21 December 2018 [2]. The Food and Agriculture Organization (FAO) stipulates the maximum residue limits (MRL) value of fipronil in different tissues, among which, the lowest MRL in poultry muscles and eggs should not exceed 0.02 mg/kg, which is consistent with China’s regulations [2]. In the EU and Japan, the maximum MRL values of fipronil are 0.005 and 0.002 mg/kg [7], respectively, which are far lower than that of FAO.

At present, many detection standards related to residual standards of fipronil are applied to some plant-derived products and honey. The existing methods for detecting fipronil mainly include gas chromatography-mass spectrometry (GC-MS), liquid chromatography-mass spectrometry (LC-MS), and gas chromatography (GC) [8,9,10,11]. The decision limits of fipronil in eggs and muscle matrices are 0.002 µg/kg and 0.001 µg/kg, respectively, by liquid chromatography-mass spectrometry/mass spectrometry (LC-MS/MS) analysis [12]. A quantitative LC/UV/MS/MS method was developed and successfully used in quantifying fipronil and fipronil sulfone in rat plasma at concentrations ranging from 2.5 to 2500 ng/mL [13]. An LC-MS/MS method for the simultaneous analysis of fipronil in okra was reported, and the limit of quantification (LOQ) is 1 ng/g for fipronil [14]. The methods for the determination of fipronil residues in eggs principally involve Gas chromatography-electron bombardment ion source-mass spectrometry (GC-EI-MS) [15], gas chromatography-triple quadrupole mass spectrometry [16], LC-MS/MS [17], ultra-performance liquid chromatography-quadrupole-linear ion trap mass spectrometry (UPLC-QqLIT-MS/MS) [18], quenched electrochemiluminescence sensor [19], etc. Recently, a method with Raman microscopy to detect fipronil in eggshells and liquid eggs was reported [2]. Although this method needs a short detection time, the limit of detection (LOD) (0.32 mg/kg) is higher than other methods.

Raman spectroscopy is a spectral method for studying the vibration of the molecule. However, it is difficult to determine pesticide residues below one part-per-million scales without any enhancement because of the weak spontaneous Raman scattering. The intensity of Raman scattering signals can be increased by several orders of magnitude in SERS analysis because Raman scattering of molecules will be greatly enhanced when the molecule deposits on or near a suitable surface with plasma activity, such as a rough nanostructured metal surface or metal colloid [20]. Various SERS-activated substrates have been widely studied in biology, as well as analytical and medical fields, and exhibit good SERS performance [21]. However, the particle size, shape, and composition influence deeply the effect of SERS enhancement. Nanomaterials such as flower-shaped [21], spiny [22], and dendritic nanoparticles [23], provide abundant sites for target molecule based on plenty of “hot spots”. Under the action of substrates with plenty of “hot spots”, the enhancement factor for SERS can be as high as 10^14^~10^15^ [24]. Currently, as a sensitive technology, SERS has been widely used in surface science, materials science, biomedicine, trace analysis and detection [25,26,27,28]. In this paper, using SERS technology and a new type of AuNPs as the substrate, we developed a simple but sensitive method for detecting fipronil residue in eggshells.

## 2. Materials and Methods

### 2.1. Materials

Raw eggs were purchased from a local farmer’s market of Tai’an, China. The analysis was conducted in advance to ensure that the eggs were not polluted by the target compound. All the chemical reagents in this work were of analytical grade and used directly without further process. The water used in this experiment was double-distilled.

### 2.2. Synthesis of Surfactant

The Bola cationic surfactant was prepared as per a previously reported method [29]. 1-Methylpyrrolidine and 1, 10-dibromodecane at a mole ratio of 2:1 were added to the acetone at room temperature (25 °C). After stirring for 30 min at 25 °C, the mixture was refluxed for 48 h at 80 °C. All the procedures were carried out in the dark. After being cooled to room temperature, the mixture obtained from the above process was filtered and recrystallized from ethanol/ether to obtain a white product.

### 2.3. Preparation and Characterization of AuNPs

Multibranch AuNPs were prepared according to the method of the previous reports [30]. Chloroauric acid (110 μL, 0.048 mol/L) and surfactant solution (230 μL, 0.1 mol/L) were added to 10 mL double-distilled water at room temperature, then ascorbic acid (750 μL, 0.1 mol/L) was added. After incubated for 12 h, the mixture was centrifuged at 8000× *g* and washed three times by ultrasonic concussion. Spherical AuNPs were prepared with a reduction of chloroauric acid by sodium citrate. Briefly, 2 mL of 1% trisodium citrate was added to 100 mL of 1% chloroauric acid, which had been boiled. The mixture was heated sequentially until the solution turned a burgundy color. After stopping being heated, the mixture was centrifuged at 8000× *g* and washed three times with water by ultrasonic concussion.

Transmission electron microscopy (TEM; JEM-1400, JEOL Ltd., Tokyo, Japan) and scanning electron microscopy (SEM, Regulus8100, HITACHI, Tokyo, Japan) were used to characterize the images and particle size of AuNPs. Fabrication of samples for TEM observation was performed by dropping preprepared solution onto 300-mesh carbon-coated copper grids. Silicon wafers with preprepared solution were used for SEM observation. The SmartLab SE diffractometer (Cu Kα radiation, λ = 0.154 nm) was selected to perform X-ray diffraction (XRD) analysis. 

### 2.4. Sample Preparation

Rhodamine 6G (R6G) stock solution of 1 × 10^−4^ mol/L was prepared by dissolving R6G powder in double-distilled water. A series of R6G solutions with concentrations from 1 × 10^−10^ to 1 × 10^−5^ mol/L was prepared by diluting the R6G stock solution with double-distilled water.

For the detection of fipronil, a stock solution at the concentration of 1.5 g/L was prepared by dissolving 150 mg of fipronil powder in 100 mL acetone. A series of fipronil solutions with concentrations from 1 pg/L to 1 g/L were prepared by diluting the stock solution using acetone. All the prepared solutions were placed at 4 °C in the dark for later use, and all the processes were carried out in the dark.

The samples for the reproducibility experiment were prepared by dropping 1 × 10^−8^ mol/L R6G or 100 ng/L fipronil onto AuNPs respectively and then blow-dried. 

For establishing the standard curve of the fipronil solution, samples were prepared by dropping 10 μL of fipronil solution with different concentrations onto AuNPs that were dropped onto glass slides or the eggshells in advance.

For the recovery experiment of fipronil on eggshells, 0.8 mL of the fipronil acetone solution with different concentrations of 100, 10, 1 µg/L were carefully dropped onto the blank eggshells which were cut into ten-centimeter squares. After drying in darkness, 0.8 mL of acetone containing AuNPs was dropped on eggshells polluted by fipronil. As to the establishment of the calibration curve, AuNPs was pretransferred evenly on the surface of eggshells in an area of 10 cm^2^, and 0.8 mL fipronil solution with different concentrations were dropped onto AuNPs. After being blow-dried, the SERS spectra were collected directly.

For the recovery experiment of fipronil from egg liquids, 5 kg of eggs were broken, and the whole liquid was collected and homogenized for 5 min. The homogenate (5 g) was vortex-mixed with 10 mL of fipronil acetone solution for 1 min and then sonicated for 10 min at 25 °C. Then, the mixture was incubated at −20 °C for 15 min. Thereafter, 4 g of anhydrous magnesium sulfate and 1 g of sodium chloride were added, and the mixture was shaken for 2 min, and then centrifuged at 7000× *g*, 4 °C for 10 min. The supernatant was decanted carefully, and the residue was re-extracted with 10 mL of acetone. The mixture was then centrifuged at 7000× *g*, 4 °C for 10 min, and the supernatant was merged with the previous supernatant. Next, 15 mL supernatant was evaporated to 5 mL at 40 °C under a stream of nitrogen. The remaining solutions were kept at −80 °C for 30 min for freeze degreasing. Finally, the supernatants after freeze degreasing were dropped onto AuNPs. After being blow-dried, the SERS spectra were collected directly.

### 2.5. SERS Measurement

The formation and distribution of fipronil crystals could not be controlled easily and it presents a challenge when minimizing the signal variation. Using the Raman microscope to observe the crystals helped in minimizing the signal variation by selecting the detected spot manually which were representative of the total population.

SERS spectra were recorded using a laser confocal microscopy Raman spectrometer (XploRA™ PLUS, Horiba, France). The excitation source was tuned at 638 nm, and the acquisition time was 10 s. A 50× objective was selected for spectral acquisition.

### 2.6. Statistical Analysis Energy

Each experiment was in triplicate to ensure the reliability of the methods, and the data were presented as a mean value with its standard deviation (SD).

### 2.7. The Assignment of Fipronil Raman Bands

As a computing method for studying the electronic structure, density functional theory (DFT) was previously proved to perform very well both for geometry optimizations and many other molecular properties [31]. Therefore, the molecular geometry optimization and vibrational spectra calculations in this experiment were studied with the DFT method.

For interpreting the Raman spectral information of fipronil, DFT calculation was carried out [32]. The basis sets used for C, N, S, O, H, F, Cl atoms in this work were 6-311 + G (d,p). The geometries were fully optimized without any constraint on the geometry and the optimized structures had no imaginary frequencies. 

## 3. Results and Discussion

### 3.1. Characterization of AuNPs

**Figure 1:**
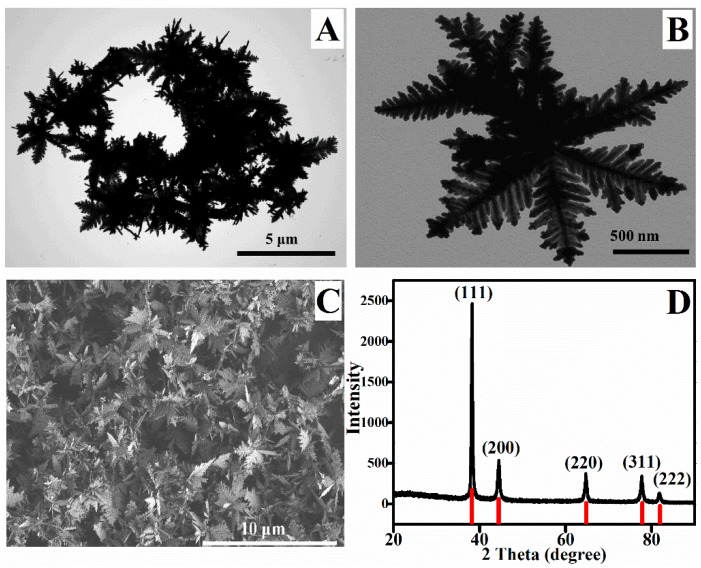
(**A**) Typical TEM image, (**B**) Enlarged TEM image, (**C**) Typical SEM image, and (**D**) XRD pattern of multibranch AuNPs.

The structural information of the obtained multibranch AuNPs was analyzed with TEM and XRD. After 12 h of growth, the AuNPs with almost entirely of multibranch structures were obtained (Figure 1A,B). The SEM image in Figure 1C shows that all of the sample spots were covered completely, and the AuNPs were in the same shape consisted of several branches with leaves and each branch was symmetric with a plane of them and associated with sharp edges and tips. 

As is known, the anisotropic growth of metal crystals is a complex process with the participation of the growth kinetics process. Anisotropic growth adjusts the shape and results of crystals in the kinetic control of the growth rate for the different facets [33,34]. A slow reduction of Au^3+^ generates the nucleation, followed by particle growth with kinetics control, and then results in the formation of stable, faceted particles and preserves the lowest surface energy of the main facet [35]. The XRD pattern exhibited the sharp and high diffraction peaks, which represents typical crystalline solids (Figure 1D). Five main diffraction peaks at 2θ = 38.25°, 44.50°, 64.72°, 77.68° and 81.76° associating with the Au (111), (200), (220), (311) and (222) planes could be indexed (PDF card 03-065-8601). It was noticeable that the intensity ratio (111)/(200) of multibranch AuNPs (=4.6) (Figure 1D) and spherical AuNPs (=3.2) (Figure S1B) were both higher than the value for standard diffraction of Au crystals (=2.6). It might be owing to the quick deposition of metallic gold along the (111) facet whose surface energy was lowest among all the facets [36]. Moreover, when growing in the shape of multibranch, metallic gold deposits along the (111) facet more quickly than that of spherical AuNPs, which indicated the larger proportion of the (111) facet of multibranch AuNPs. The size of individual multibranch AuNPs was about 1.5 μm (Figure 1B), and the diameter of spherical AuNPs was distributed between 10 to 20 nm (Figure S1A). The dendritic structure of multibranch AuNPs exhibited symmetrical planar structure with many groups of parallel branches and apexes, all of which could serve as “hot spots” to enhance the Raman signals and provide more sites for the fipronil molecules. When molecules stopped close to “hot spots”, strong SERS signal of fingerprints would be generated [37,38].

### 3.2. SERS Enhancement and Uniformity of the AuNPs

Using R6G as a probing molecule, the SERS activity of the AuNPs was studied. The Raman spectrum of solid R6G exhibited very weak signals. However, spectral signals of R6G solution droplets on the multibranch AuNPs substrate were enhanced, and the SERS spectra exhibited sharp peaks (Figure S2A). 

Indeed, the characteristic signal of R6G was significantly enhanced when R6G was dropped onto the multibranch AuNPs. Compared to solid R6G, R6G@AuNPs caused the intense SERS peaks, especially at 610, 770, 1178, 1305, 1357, 1504, and 1643 cm^−1^ (Figure S2A). The peaks at 610, 770, and 1178 cm^−1^ attribute to C-C-C ring in-plane vibration, C-H out-of-plane vibration, and C-H in-plane vibration, respectively [39]. The peaks at 1305, 1357, 1504, and 1643 cm^−1^ are corresponding to in-plane xanthene ring breathing, xanthene ring stretching with aromatic C-C stretching, in-plane C-H bending, and xanthene ring stretching with in-plane N-H vibration, respectively [40]. These peaks could still be observed even when the R6G solution was diluted to 1 × 10^−8^ mol/L (Figure S3A). However, the characteristic peaks of R6G were not obvious at 1 × 10^−7^ mol/L when spherical AuNPs were used as the substrate (Figure S3B). Thus, compared with the spherical nanogold, multibranch AuNPs as the substrate could enhance the SERS signal of R6G better.

R6G was used as a Raman probe molecule for its well-established vibrational features. Therefore, SERS spectra of 1 × 10^−8^ mol/L R6G@multibranch AuNPs were randomly collected on 10 substrates, and all of them exhibited the same shapes (Figure S2B). The peak at 1357 cm^−1^ with the strongest intensity was selected to examine the uniformity of these substrates. The intensity statistics of R6G Raman peak at 1357 cm^−1^ and the relative standard deviation (RSD) (n = 10) was 8.75% (Figure S2C). The satisfactory RSD values indicated that this substrate expressed good uniformity in the SERS experiment.

### 3.3. Practical Application for Sensing Fipronil

On account of its high SERS activity, the multibranch AuNPs were used as the substrate to detect fipronil. From Figure S4, the detection spot were selected manually by using the Raman microscope in order to minimize the signal variation, resulting in a more consistent signal intensity.

Because of a large number of “hot spots” in multibranch AuNPs, the Raman signal of fipronil could be immensely enhanced. The SERS spectra of the pure multibranch AuNPs-substrate and fipronil without substrate were smooth lines without obvious vibration peaks. 

**Figure 2:**
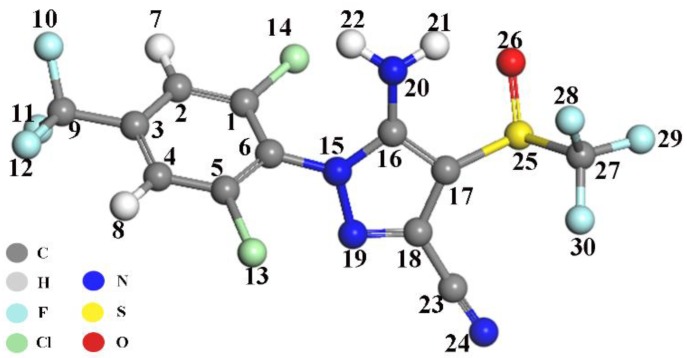
Fipronil structure with the numbered atom.

**Figure 3:**
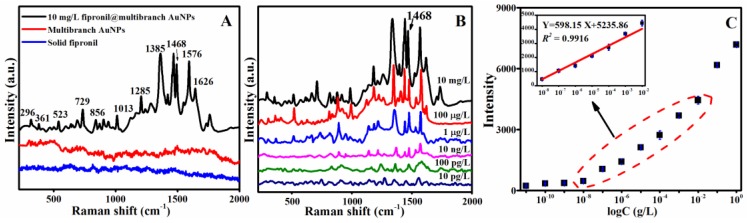
(**A**) SERS spectra of 10 mg/L fipronil at multibranch AuNPs, multibranch AuNPs, and solid fipronil. (**B**) SERS spectra of fipronil at multibranch AuNPs at different concentrations. (**C**) Relationship plot between peak intensity at 1468 cm^−1^ and log C.

Raman spectroscopy provided detailed structural information, and for the purpose of applying the SERS method in fipronil detection, the experimental results of the SERS bands for solid fipronil were assigned and distributed according to the simulation results of DFT calculation and previous report [31,41]. The optimized structural representation of fipronil in its neutral form is shown in Figure 2, while after the addition of fipronil on multibranch AuNPs-substrate, the most significant SERS bands were observed at 296 cm^−1^ [β(F_12_-C_9_-F_11_) + γ(F_10_-C_3_-F_12_-C_9_)], 361 cm^−1^ [δ(N_24_-C_23_-C_18_-C_17_) + δ(C_17_-C_16_-N_15_-C_6_) + δ(C_18_-N_19_-N_15_-C_6_) + ν(C_3_-C_2_)], 523 cm^−1^ [υ(C_23_-C_18_) + β(C_18_-N_19_-N_15_)], 729 cm^−1^ [δ(C_18_-N_19_-N_15_-C_6_) + γ(C_23_-C_17_-N_19_-C_18_) + ν(C_3_-C_2_)], 856 cm^−1^ [β(C_4_-C_3_-C_2_), ν(S_25_-O_16_)], 1013 cm^−1^ [ν(C_23_-C_18_) + β(C_17_-C_16_-N_15_) + β(C_16_-N_15_-N_19_)], 1285 cm^−1^ [υ(C_9_-C_3_) + β(H_7_-C_2_-C_3_) + β(H_8_-C_4_-C_5_) + ν(C_17_-C_16_)], 1385 cm^−1^ [ν(N_19_-C_18_) + β (C_18_-N_19_-N_15_) + β(C_16_-N_15_-N_19_)], 1468 cm^−1^ [υ(C_23_-C_18_) + β(C_18_-N_19_-N_15_) + β(C_17_-C_16_-N_15_) + β(C_16_-N_15_-N_19_)], 1576 cm^−1^ [υ(N_15_-C_16_) + υ(N_15_-C_6_)] and 1626 cm^−1^ [υ(C_17_-C_16_) + υ(N_20_-C_16_) + β(H_22_-N_20_-H_21_)] (Figure 3A). Those significant SERS bands should be attributed to fipronil characteristic vibration peaks. Vibration peaks of fipronil could be distinguished from reference even if the fipronil concentration was diluted to 10 pg/L (Figure 3B). However, vibration peaks could not be observed at fipronil concentration of 1 µg/L, when spherical AuNPs were used as the substrate (Figure S6). The enhancement factor (EF) was introduced and calculated with Formula (1) [36]:

EF = (I_SERS_/I_bulk_)(N_bulk_/N_SERS_) (1)

In which I_SERS_ is the intensity of SERS spectra of the sample, I_bulk_ is the normal Raman spectra of fipronil, N_SERS_ is the total number of surface adsorbed molecules, and N_bulk_ is the number of molecular in the laser illumination volume. The mean value of EF for multibranch AuNPs is as high as 9.04 × 10^5^, while for spherical AuNPs is only 572.88. All of the results indicated that multibranch AuNPs performed much more sensitive to detect fipronil than spherical AuNPs. In addition, as expected, multibranch AuNPs offered simplicity and high sensitivity for the real application.

Amongst these characteristic fipronil vibration peaks, the SERS peak at 1468 cm^−1^ was selected for quantitative analysis because of the relatively strong intensity which was able to be observed even when the concentration was very low. The peak intensity had a good linear relationship with logarithm of fipronil concentration (log C) in the wide range of low concentration from 10 ng/L to 10 mg/L (Figure 3C), with excellent correlation (*R*^2^ = 0.9916). The calculated limit of detection (LOD_0_) and the calculated limit of quantity (LOQ_0_) [42] for the proposed method were 4.49 ng/L and 13.61 ng/L, respectively, which were determined according to International Council for Harmonization (ICH) guidelines [43,44] based on the standard deviation of the response (σ) and the slope of the calibration curve (S), and were generally defined as:

(2) $${\mathrm{LOD}}_{0}\;=\;3.3\;\sigma /\;S$$

(3) $${\mathrm{LOQ}}_{0}\;=\;10\;\sigma /\;S$$

Since reproducibility is essential for quantitative SERS measurements, the experiment was conducted to determine the reproducibility of the measurement with AuNPs loaded by 100 µg/L fipronil. Spectra of 100 µg/L fipronil at multibranch AuNPs behaved in a similar manner (Figure S5A), and the RSD was 8.5% at the peak of 1468 cm^−1^ (Figure S5B). These investigations supported a high reproducibility of SERS intensity and revealed the potential of the introduced method to detected fipronil.

Although forbidden to use, fipronil might be illegally interpolated into eggs due to its high insecticidal activity. In this study, the SERS method with the obtained multibranch AuNPs as substrate was also utilized for fipronil detection from eggshells and egg liquids.

**Figure 4:**
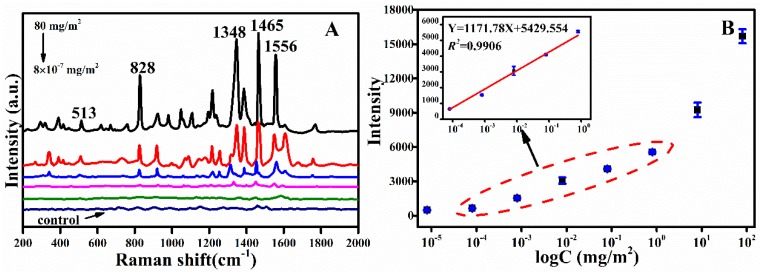
(**A**) SERS spectra of fipronil at multibranch AuNPs at different concentrations on eggshells (80, 0.8, 8 × 10^−3^, 8 × 10^−5^, 8 × 10^−6^ mg/m^2^). (**B**) Relationship plot between peak intensity at 1468 cm^−1^ and log C.

The SERS spectra of fipronil with incremental concentrations from 8 × 10^−7^ to 80 mg/m^2^ using the AuNPs on eggshells as the substrate were collected (Figure 4A). One can find that the Raman peaks got intense, obviously as the fipronil concentration increased. Raman bands remained discernible even when the fipronil concentration decreased to 8 × 10^−6^ mg/m^2^. However, when the fipronil concentration was 8 × 10^−7^ mg/m^2^, the Raman signals could not be monitored. In Figure 4B, a good linear relationship could be obtained in the wide low concentration from 8 × 10^−5^ mg/m^2^ to 0.8 mg/m^2^ with *R*^2^ of 0.9906. LOD of fipronil residue on eggshells was calculated to be 7.59 × 10^−5^ mg/m^2^, and the LOQ was 2.30 × 10^−4^ mg/m^2^. In addition, the recovery rates on eggshells were also calculated to evaluate the method accuracy. The recovery rate was calculated using the Formula (4):

(4) $$\mathrm{Recovery}\;\mathrm{rate}\;(\text{\%})\;=\;\frac{C_{1}}{C_{0}}\;\times \;100\text{\%}$$

In Formula (4), C_0_ is the original concentrations of fipronil on eggshells, and C_1_ is the concentration of the recovering solution that could be confirmed according to the calibration curve as shown in Figure 4B. The calculated recovery rate of the analyte spiked at three concentration (C_0_) levels of 8 × 10^−2^ mg/m^2^, 8 × 10^−3^ mg/m^2^ and 8 × 10^−4^ mg/m^2^, which were 87.87% (RSD = 10.17%), 80.37% (RSD = 7.25%), and 80.13% (RSD = 8.52%), respectively. 

To transform the LOD_0_ and LOQ_0_ in the standard curve of fipronil acetone solution to the number that is based on the weight of egg liquids, Equations (5) and (6) was used. Theoretical LOD of fipronil in egg liquids was 8.98 ng/kg, and the LOQ was 26.94 ng/kg. In Formulas (5) and (6), LOD_0_ and LOQ_0_ are the limit of detection and the limit of quantity, respectively, as calculated by Formulas (2) and (3).

(5) $$\mathrm{LOD}\;=\;\frac{{\mathrm{LOD}}_{0}\;\times \;10\;\mathrm{mL}}{m}$$

(6) $$\mathrm{LOQ}\;=\;\frac{{\mathrm{LOQ}}_{0}\;\times \;10\;\mathrm{mL}}{m}$$

In addition, the recovery rates of egg liquids were calculated using Formula (4). When calculating the recovery rates of egg liquids, C_0_ would be the original concentrations of fipronil added into egg liquids, and C_1_ would be the concentration of the recovering solution that could be confirmed according to the calibration curve as shown in Figure 3C. The recovery rate of the analyte spiked at three concentration (C_0_) levels of 200, 20, and 2 µg/kg was 88.89% (RSD = 8.83%), 85.76% (RSD = 5.00%), and 81.34% (RSD = 5.99%), respectively. These results demonstrated that the SERS method with multibranch AuNPs as the substrate was more compatible with many previous analytical methods for fipronil detecting in eggs (Table S1), and would be more sensible than recently reported strategies of SERS with various other substrates [2,32,41]. 

**Figure 5:**
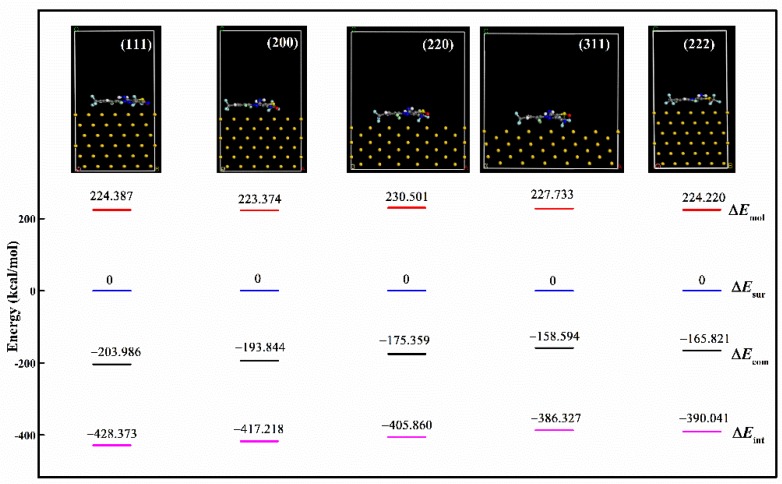
Molecular dynamics simulation results of fipronil molecules on different gold crystal surfaces. Blue, gray, white, red, aqua, wathet, and gold represent N, C, H, O, Cl, F, and Au, respectively. (Δ*E*_int_: The interaction energies of fipronil molecules and AuNPs; Δ*E*_com_: Total energy of complex; Δ*E*_mol_: The energy of fipronil molecule; Δ*E*_sur_: The energy of the constrained gold surface.).

For assessing the advantage of crystals with preferential (111) orientation in SERS sensing of fipronil, the molecular dynamic simulations for fipronil on the Au surface were completed (Figure 5). Five lattice planes were involved, including Au(111), (200), (220), (311), and (222). The compass force field existed in nonbonded interactions of both intermolecular and intramolecular interactions. Based on the compass force field, the interaction energy (Δ*E*_int_) was calculated from the difference between the energy of complex (Δ*E*_com_), fipronil molecules (Δ*E*_mol_) and gold surface (Δ*E*_sur_) with the following Formula (7):

Δ*E*_int_ = Δ*E*_com_ − Δ*E*_mol_ − Δ*E*_sur_ (7)

On the Au(111) surface, the interaction energy was −428.373 kcal/mol, while that of Au(200), Au(220), Au(311), and Au(222) was –417.218, –405.860, –386.327 and −390.041 kcal/mol, respectively (Figure 5). It was obvious that the fipronil molecule was preferentially deposited on Au(111) because of the lowest interaction energy of Au(111). Therefore, with Au(111) as the predominant facet, multibranch AuNPs were expected to be a promising SERS substrate for the effective detection of fipronil.

## 4. Conclusions

With abundant “hot spots”, multibranch AuNPs can effectively enhance fipronil SERS signals. Along with a wide linear response, the LOD for fipronil on eggshells and in egg liquids were better than many analytical methods for fipronil detection, and the recoveries were satisfactory. As a simple and highly sensitive approach for fipronil detecting, the SERS method was successfully applied to detect fipronil from eggshells and egg liquids.

## Supplementary Materials

The following are available online at https://www.mdpi.com/1424-8220/19/24/5354/s1, Figure S1: (A) Typical TEM images of the spherical AuNPs. (B) XRD pattern of spherical AuNPs. Figure S2: (A) Raman spectrum of solid R6G and SERS spectrum of 1×10^−8^ mol/L R6G@AuNPs. (B) Ten sequential SERS spectra of 1 × 10^−8^ mol/L R6G@multibranch AuNPs. (**C)** Intensity statistics of R6G Raman peaks at 1357 cm^−1^. Figure S3: (A) Average SERS spectra of R6G@multibranch AuNPs at different concentrations (1 × 10^−10^~1 × 10^−7^ mol/L). (B) Average SERS spectra of R6G@spherical AuNPs at different concentrations (1 × 10^−8^~1 × 10^−5^ mol/L). Figure S4: Photograph of fipronil sample (10 mg/L in acetone) on multibranch AuNPs. Figure S5: (A) Ten sequential SERS spectra of 100 ng/L fipronil absorbed on multibranch AuNPs substrate. (B) Intensity statistics of fipronil Raman peak at 1468 cm^−1^. Figure S6: SERS spectra of fipronil solution@spherical AuNPs with different concentrations (10 ng/L~1.2 mg/L), Table S1: Comparison of the response characteristics between this SERS assay and some other studies about fipronil detection in eggs published in the literature.
