# Supplementary material for: Multibranch Gold Nanoparticles as Surface-Enhanced Raman Spectroscopy Substrates for Rapid and Sensitive Analysis of Fipronil in Eggs
Source: Sensors (Basel). 2019 Dec 5;19(24):5354. doi: 10.3390/s19245354 (PMC6960814; doi:10.3390/s19245354)
Supplement: Supplementary file 1 [file sensors-19-05354-s001.zip › sensors-654146-supplementary-final/R2-Supplementary files-sensors-654146/R2-Supplementary Files-sensors-654146.docx]

SUPPLEMENTARY MATERIAL FOR

Multibranch Gold Nanoparticles as Surface-Enhanced Raman Spectroscopy Substrates for Rapid and Sensitive Analysis of Fipronil in Eggs

Haonuan Zhao ^1^, Dandanhuang * and Shuhua Zhu *

College of Chemistry and Material Science, Shandong Agricultural University, Tai’an 271000, China; [zhaohaonuan@163.com](mailto:zhaohaonuan@163.com) (H.Z.)

***** Correspondence: [ddhuang@sdau.edu.cn](mailto:ddhuang@sdau.edu.cn) (D.H.); [shuhua@sdau.edu.cn](mailto:shuhua@sdau.edu.cn) (S.Z.)

# Supplementary Figures





Figure S1: (A) Typical TEM images of the spherical AuNPs. (B) XRD pattern of spherical AuNPs.





Figure S2: (**A)** Raman spectrum of solid R6G and SERS spectrum of 1×10^-8^ mol/L R6G@AuNPs. (**B)** Ten sequential SERS spectra of 1×10^-8^ mol/L R6G@multibranch AuNPs. (**C)** Intensity statistics of R6G Raman peaks at 1357 cm^-1^.

**

**

## Figure S3: (A) Average SERS spectra of R6G@multibranch AuNPs at different concentrations (1×10^-10^ ~ 1×10^-7^ mol/L). (B) Average SERS spectra of R6G@[spherical](javascript:;) AuNPs at different concentrations (1×10^-8^ ~ 1×10^-5^ mol/L).

**
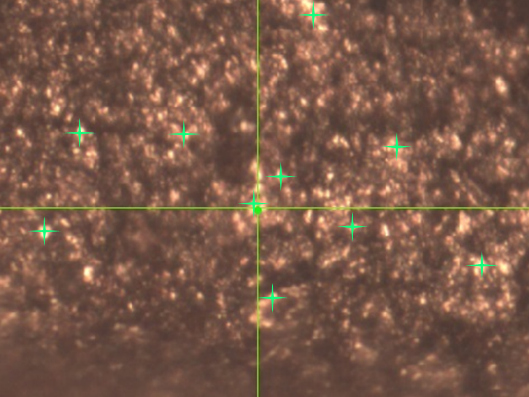
**

Figure S4: Photograph of fipronil sample (10 mg/L in acetone) on multibranch AuNPs.





Fig**ure S5:** (A) Ten sequential SERS spectra of 100 ng/L fipronil absorbed on multibranch AuNPs substrate. (B) Intensity statistics of fipronil Raman peak at 1468 cm^-1^.





Figure **S6:** SERS spectra of fipronil solution@spherical AuNPs with different concentrations (10 ng/L ~ 1.2 mg/L).

# Supplementary Tables

# Table 1. Comparison of the response characteristics between this SERS assay and some other studies about fipronil detection in eggs published in the literature.

| **Method** | **LODs** | **Recovery rate (%)** | **RSD (%)** | **References** |
| --- | --- | --- | --- | --- |
| GC-EI-MS | 0.2 - 0.5 ng/g | 81.16 - 103.57 | 3.32 | [1] |
| Electrochemiluminscence sensor | 1.5 nmol/L | 95.6 - 104.6 | < 5 | [2] |
| Raman microscopy | 0.32 mg/kg | 85.97 - 152.46 | < 10 | [3] |
| SERS  (with SiO_2_@Au core/shell) | 10^-7^ mol/L | —— | —— | [4] |
| SERS  (with multi-branch AuNPs) | 75.9 ng/m^2^ (eggshells) | 80.13 - 87.87 | 7.25 – 10.17 | This work |
|  | 51.57 ng/kg (egg liquids) | 81.34 - 88.89 | 5.00 – 8.83 |  |

# References

1. Li, X.; Li, H.; Ma, W.; Guo, Z.; Li, X.; Song, S.; Tang, H.; Li, X.; Zhang, Q., Development of precise GC-EI-MS method to determine the residual fipronil and its metabolites in chicken egg. *Food Chem.* **2019,** 281, 85-90.

2. Yin, J.; Chen, X.; Chen, Z., Quenched electrochemiluminescence sensor of ZnO@g-C3N4 modified glassy carbon electrode for fipronil determination. *Microchem. J.* **2019,** 145, 295-300.

3. Tu, Q.; Hickey, M. E.; Yang, T.; Gao, S.; Zhang, Q.; Qu, Y.; Du, X.; Wang, J.; He, L., A simple and rapid method for detecting the pesticide fipronil on egg shells and in liquid eggs by Raman microscopy. *Food Control* **2019,** 96, 16-21.

4. Muhammad, M.; Yao, G.; Zhong, J.; Chao, K.; Aziz, M. H.; Huang, Q., A facile and label-free SERS approach for inspection of fipronil in chicken eggs using SiO_2_@Au core/shell nanoparticles. *Talanta* **2020,** 207, 120324.
