# Supplementary figures and images for: Multibranch Gold Nanoparticles as Surface-Enhanced Raman Spectroscopy Substrates for Rapid and Sensitive Analysis of Fipronil in Eggs
Source: Sensors (Basel). 2019 Dec 5;19(24):5354. doi: 10.3390/s19245354 (PMC6960814; doi:10.3390/s19245354)

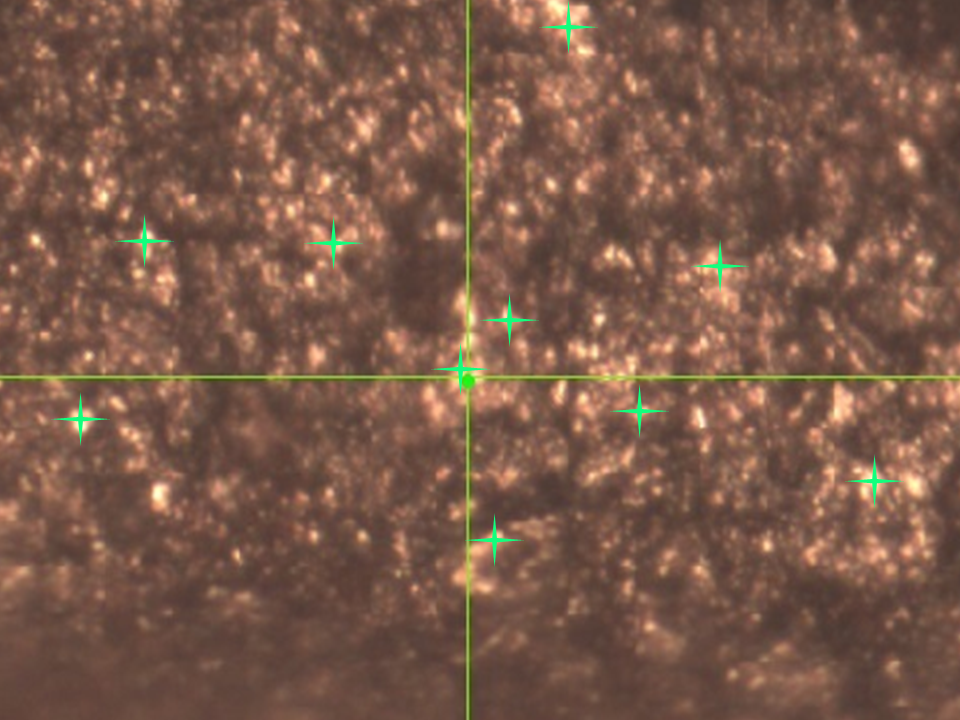

Supplement: Supplementary file 1 [file sensors-19-05354-s001.zip › sensors-654146-supplementary-final/R2-Supplementary files-sensors-654146/Figure S4.tif]
